# Machine Learning Approaches: From Theory to Application in Schizophrenia

**DOI:** 10.1155/2013/867924

**Published:** 2013-12-09

**Authors:** Elisa Veronese, Umberto Castellani, Denis Peruzzo, Marcella Bellani, Paolo Brambilla

**Affiliations:** ^1^Scientific Institute IRCCS “Eugenio Medea”, San Vito al Tagliamento, 33078 Pordenone, Italy; ^2^Department of Informatics, University of Verona, 37134 Verona, Italy; ^3^Scientific Institute IRCCS “Eugenio Medea”, Bosisio Parini, 23842 Lecco, Italy; ^4^Department of Public Health and Community Medicine, Section of Psychiatry and Section of Clinical Psychology, ICBN, University of Verona, 37134 Verona, Italy; ^5^Department of Experimental & Clinical Medical Sciences (DISM), ICBN, University of Udine, 33100 Udine, Italy; ^6^Department of Psychiatry and Behavioral Sciences, UT Houston Medical School, Houston, TX 77054, USA

## Abstract

In recent years, machine learning approaches have been successfully applied for analysis of neuroimaging data, to help in the context of disease diagnosis. We provide, in this paper, an overview of recent support vector machine-based methods developed and applied in psychiatric neuroimaging for the investigation of schizophrenia. In particular, we focus on the algorithms implemented by our group, which have been applied to classify subjects affected by schizophrenia and healthy controls, comparing them in terms of accuracy results with other recently published studies. First we give a description of the basic terminology used in pattern recognition and machine learning. Then we separately summarize and explain each study, highlighting the main features that characterize each method. Finally, as an outcome of the comparison of the results obtained applying the described different techniques, conclusions are drawn in order to understand how much automatic classification approaches can be considered a useful tool in understanding the biological underpinnings of schizophrenia. We then conclude by discussing the main implications achievable by the application of these methods into clinical practice.

## 1. Introduction

Investigating the neurobiological bases of psychiatric disorders requires a large sample studied in a longitudinal perspective from early stages of the diseases. In this context, magnetic resonance imaging (MRI) is the gold-standard technique to explore the anatomical and functional underpinnings of such illnesses [1–3].

In order to accurately analyze such large amount of imaging data, automated methods are becoming essential [4]. As outlined by Lao and colleagues [5], to develop an accurate detector of pathology from a set of images, two issues need to be addressed. First, an image analysis methodology is needed in order to extract the most relevant information from the images. Second, a pattern classification method has to be designed to process the extracted information, in order to determine the likelihood of the disease.

*Feature extraction* is aimed at characterizing an object in terms of properties, or *features*, such as dimensions, shape, color, and texture. Chosen features are those that, when belonging to objects of the same category, or *class*, are very similar; on the contrary, they should be very different from objects in different categories. The set of features extracted from an object can be considered as a signature which describes the object itself. Features are usually organized in the so-called *feature vector*, a vector of arbitrary length which collects all the properties that are considered useful in order to describe the objects under analysis. A good feature vector should be able to discriminate objects belonging to different classes.

*Classification* is aimed at finding a rule that, based on the available features, distinguishes all the objects and assigns them to the specific category.

These two issues are typically referred to as *feature extraction* and *classification*. Particularly, the aim of *feature extraction* is to characterize an object in terms of features (such as dimensions, shape, color, and texture) whose values should be on one hand very similar for objects in the same category, or *class*, and, on the other hand, very different for objects in different categories. The set of the features extracted from an object can be considered as a signature which describes the object itself. Features are usually organized in the so-called *feature vector*.

*Pattern recognition* is the science which uses statistics and mathematics to program a computer in order to recognize patterns in a dataset. In the field of medical science, pattern recognition is the basis of computer aided diagnosis (CAD) systems. Other fields of application include, for instance, fingerprint identification, automatic speech recognition, DNA sequence identification, and so on.

The aim of *classification*, instead, is to find a rule that, based on the available features, distinguishes all the objects and assigns them to the specific category.

The problems of features extraction and classification are standard issues in the field of computer vision and artificial intelligence. We use the term *pattern recognition *to identify the science which employs statistics and mathematic tools to teach a computer to recognize patterns in a data set. In the field of medical science, pattern recognition is the basis of computer aided diagnosis (CAD) systems. Among the set of tools, *machine learning *refers to the ability of a system to change its behavior without being explicitly programmed. It is linked to artificial intelligence, and it allows computers to handle new situations by means of previous experience, analysis, and self-training. In machine learning the classification aims at automatically identifying to which of a set of classes a new observation belongs.

*Machine learning* is linked to artificial intelligence, and it designs algorithms in order to allow a computer to learn from data. In this context, the term *learn *means finding statistical regularities on a set of data. Machine learning allows computers to handle new situations by means of previous experience, analysis, and self-training. The ability of machine learning in turning data into information is exploited in problems of pattern recognition. For example, spam email can be automatically detected by looking, for instance, at the occurrence of a set of words in the object and in the body of the email, combined with the length of the text, the presence of attached files, and so forth.

In particular, features in the data are automatically searched, in order to use them to classify the data into different predefined classes. This is done on the basis of a set of data containing observations of which class membership is known, called *training set*. A classifier is any algorithm that implements classification. Typically a classifier takes the values of various features of an instance to be classified and, exploiting the provided training set, predicts to which class the instance belongs. As summarized in Pereira et al. [6], a classifier has a number of parameters that have to be learned from the training set. This step of *learning *makes the classifier a model of the relationship between the features and the class label in the training set. Once trained, the classifier has to be tested, in order to determine whether the selected features contain information about the class of the example or not. The performance of the classifier is tested by trying to classify a different set of examples, called *validation set*: in this way it is possible to evaluate the ability of the classifier of correctly categorizing a previously unseen instance.

A *training set* is necessary in pattern recognition in order to teach a computer to correctly classify objects, or *instances*, into classes. Given a dataset, data is split into a training set, from which a model is built, and into a *validation set*, or test set, which is used to validate the model. This model is the rule used by the computer to classify objects into classes. The training set contains instances whose class membership is known. Each instance is described by its feature vector. The training set is used by the classifier to learn which features are useful to correctly assign each instance to its class.

Once trained, the performance of the classifier can be tested by evaluating its ability to correctly classify the instances of the validation set.

In the literature, dozens of different classifiers have been proposed (support vector machines, classification trees, linear discriminant analysis, quadratic discriminant analysis, neural networks, generalized linear models, the nearest neighbor, etc.). They are all based on different algorithms, whose aim is to decide how new instances should be classified [7]. Their performance, that is, the ability of each classifier to assign new instances to their class, depends on the algorithm they analyze the data with. During the past few years, support vector machine (SVM), a supervised machine learning classifier [6, 8], has emerged as one of the most powerful pattern classification methods [5, 9], and it has become a state of the art in many classification tasks, such as object and face recognition [10], genome sequencing [11, 12], and handwritten recognition [13]. The idea at the basis of SVM is to project the feature data points to a high dimensional space where the groups can be separated using a hyperplane. Boser and colleagues [14] suggested a way to create nonlinear classifiers by an algorithm that operates with a large class of decision functions that are linear in their parameters but not restricted to linear dependences in the input components. These functions are known as *nonlinear kernel functions*.

In the 3-dimensional space, a *plane* is a 2-dimensional flat surface. Similarly, in an *n*-dimensional space, a *hyperplane* can be described as an (*n* − 1)-dimensional surface.

A *projection* is the transformation of points and lines of one plane into another plane. Corresponding points on the two planes are connected by parallel lines. An object can be projected from a space to another one of different dimensions. For instance, a sphere in a 3-dimensional space can be projected into an ellipse in a 2-dimensional space.

Any element can be projected from a space to another one of different dimensions.

In SVM, objects are projected into a high dimensional space, where they can be separated using a hyperplane which is called *decision surface* or *decision function*.

SVM has been increasingly employed in neuroimaging studies, for instance, as a multivariate method in functional MRI (fMRI) [15, 16]. In the study by Cox and Savoy [15], given a defined time point, the pattern of brain activation across space measured by fMRI was considered. The aim of the study was to learn a classifier to identify, starting from the pattern of activation, which kind of stimuli the subject was viewing (common versus uncommon objects, living creatures versus inanimate objects, etc.).

A *similarity measure* is a function to compute the degree of similarity between a pair of objects.

A *kernel function f* is used as a similarity measure; that is, two elements *x*_1_ and *x*_2_ drawn from a set *X* are considered *equivalent *if *f*(*x*_1_) = *f*(*x*_2_). In the field of pattern recognition, kernel methods are used to project data into higher dimensional space. In the new space, in fact, data could become more easily separated and classified. Different types of kernel functions are commonly used, that is, linear, polynomials, Gaussian, radial basis function (RBF), and so on.

In more detail, in a recent critical review, Orrù et al. [17] stated that using structural and/or functional neuroimaging data as input to SVM represents a valid diagnostic aid for classifying major neurological and psychiatric illnesses, allowing inferences at individual level, rather than at group level. This may ultimately have a major impact on clinical practice: as emphasized by the authors, neuroimaging can be considered useful in a clinical setting if it is not limited at reporting differences between the group of patients and the group of controls. On the contrary, it should be able to help doctors to make clinical decisions about each patient. However, the application of state-of-the-art classification methods, such as SVM, to neuroimaging field is not straightforward: clinical data has specific characteristics that pose new issues to be solved, among which are the high dimensionality of acquired brain data, the definition of features, their interpretation from the physiological point of view, the inner complexity of brain structures, and the presence of multiple covariates which contribute to the heterogeneity of a population.

In analyzing brain images, the most commonly used methods comprise the region of interest (ROI) analysis, the voxel-based morphometry (VBM) [18], and the surface-based morphometry (SBM) [19, 20]. The ROI analysis defines some regions of interest according to known a priori hypothesis and statistically analyzes some related physiological measures (e.g., their volumes). ROIs can be either manually traced by expert operators or automatically extracted by segmentation algorithms. VBM considers the whole brain after a normalization procedure which maps each subject brain onto a standard reference, namely, the stereotaxic space, allowing a voxel-by-voxel comparison with no a priori hypothesis. Finally, SBM does not analyze the image properties at voxels level, but it rather constructs and analyzes surfaces that represent structural boundaries within the brain (i.e., boundaries between white and grey matter or between grey matter and cerebrospinal fluid).

However, even though in schizophrenia structural and functional brain abnormalities in patients have been demonstrated [21, 22], neither ROI analysis, nor VMB, nor SBM techniques enable patients with schizophrenia to be automatically classified, based on the brain's features. Nonetheless, as summarized in Orrù et al. [17], during the past few years an increasing number of studies have used SVM in order to investigate the presence of potential neuroanatomical biomarkers of neurological and psychiatric disorders [23–27]. However, with the exception of the studies by Gerig and colleagues [24] and by our group [28], all the other studies applied multivariate whole brain analysis, thus being limited by the use of an immense dimensional space in a relatively small sample size. Multivariate analysis allows examining relationships among multiple variables at the same time, and it might be useful if a given outcome is hypothesized to be influenced by more than one variable. Nonetheless, results obtained using multivariate analysis can be considered meaningful only in the presence of a large dataset; otherwise, they are meaningless due to high standard errors. As for whole brain analysis, it is not always the best way to analyze changes in brain regions, since misleading significant correlations may exist in some brain regions that are not involved in the analyzed brain disease.

In addition, only the studies by Gerig and colleagues [24] and by our group [28] were driven by a priori hypothesis and consistently detected specific structural markers.

In our studies we aimed at automatically classifying schizophrenia by applying a ROI-based machine learning approach within different brain regions [36].

The main focus of this review would be, firstly, to briefly describe the principles of SVM techniques, in order to let readers unfamiliar with classification methods get acquainted with them. Successively, we will focus on our machine learning studies, comparing them in terms of accuracy results with other recently published studies. Finally, we will debate the results based on the current literature in a clinical translation perspective.

## 2. SVM Operating Principles

The working pipeline of the SVM can be divided into three separated steps: *features extraction*, *features selection*, and *classification*. In the following sections we will briefly summarize the main aspects of each of them.

### 2.1. Features Extraction

In this phase, the original data are processed in order to compute a set of representative features which can be used as input for the SVM. This is a crucial step in the SVM analysis since every measure obtained from the raw data can be ideally used as a feature for the SVM analysis; redundant or not significant features may affect the performance of the final classifier.

Feature extraction includes all procedures performed to compute some measures that characterize the object which is being investigated, for example, probability of gray matter (pGM) if we are studying the cortex using morphological images or the diffusion measures if we are studying the white matter using diffusion tensor MR images.

Features may have an intuitive physiological interpretation, such as the pGM obtained using the VBM approach [37] or not, as in the work by Selvaraj et al., [38] who used various features, among which are the energy and the entropy of the image. After features have been extracted from the data, they may undergo a normalization process to account for physiological changes which are not related to the disease, similar to what is usually done in the VBM analysis. In VBM, pGM is usually normalized to the total intracranial volume in order to account for pGM differences due to physiological differences of the brain volume among subjects. The normalization step is performed when the extracted features depend not only on the disease, but also on other physiological differences among subjects. After normalization, differences are no longer related to the total intracranial volume, and the group analysis is more robust and easy to be interpreted. If the normalization is not performed, the physiological differences may mislead the classifier, worsening its performance. Therefore, confounding factors should be eliminated. pGM can be normalized using the total intracranial volume or the total GM volume, whereas, for instance, intensity histograms can be normalized to their maximum value or to the sum of their bins.

Finally, features from each subject must be stored in a vector, that is, the *feature vector*, in order to be processed by the SVM algorithms: each two-dimensional image (or each three-dimensional volume) has to be transformed into a column vector in which each element corresponds to the gray level intensity of one pixel (or voxel, resp.). SVM analysis requires feature vectors to be of the same length. This might represent a limitation, since different subjects could be represented by a different number of features. To overcome this problem, dissimilarity vectors can be used instead of feature vectors. In this case, similarities measure is computed between each couple of subjects in the dataset and directly used as a feature in the SVM analysis; such a method is referred to as pairwise dissimilarity approach [30].

### 2.2. Features Selection

This step is optional, since the SVM algorithms do not have requirements about feature lengths. When it is performed, it reduces the variable-length sequence of observations associated with a set of extracted features. As clarified by Kloppel et al. [39], neuroimaging data can be characterized by more than one million dimensions, so a reduction of the input measures can be useful. This selection is aimed at improving the performance of classification step, since minimally important, redundant, or noisy features might worsen the discrimination between classes. Besides, a reduced number of features imply a smaller computational load, thus accelerating the overall process. Last but not least, a selection can either help or eliminate the physiological interpretability of the features provided to the classifier. In the context of neuroimaging, features selection can be performed in three ways.

1. *Filtering*. On the basis of medical knowledge, that is, exploiting a priori information, some features can be considered either noninfluential for the diagnosis of specific disease and thus can be discarded or useful for the considered disease and thus can be exploited [28, 29, 31].
2. *Before Classification*. Prior studies in the areas of feature selection and dimensionality reduction include principal component analysis (PCA) [40] or mathematical approaches such as the minimization of a concave function on a polyhedral set [41]. It should be remarked that the feature selection, performed independently of learning the classifier parameters, might result in a loss of information relevant for classification tasks. Moreover, this feature selection procedure might cause a loss of medical interpretability of the selected features.
3. *During the SVM Training*. In this case, the feature selection is embedded in the classification step. An example is represented by the sequential forward and backward selection (SFBS). In the *forward selection* [42] the process begins analyzing each feature singularly and selecting the best one. The overall process is iterative: for each step the best feature from the remaining set is selected, and the feature list and the classification performances are saved. In this case, the best feature is the feature that, combined with the already selected ones, gives the best classification results. The process is iterated until all features have been included. Finally, the feature set providing the best performance is chosen as result of the whole selection procedure. On the other way around, in the *backward selection *[43], features are progressively removed from the feature set, on the basis of some weights that the classifier assigns to each feature at each iteration. The advantage of this approach is that there is no loss in medical interpretability of the selected features; the drawback is the computational complexity since the analysis must be performed several times.

In a more general case, during the classification step, a combination of different kernel functions can be learnt, one for each feature extracted from the data. This is the case of the recently introduced multiple kernel learning (MKL) methods [33, 34].

### 2.3. Classification

**Figure 1:**
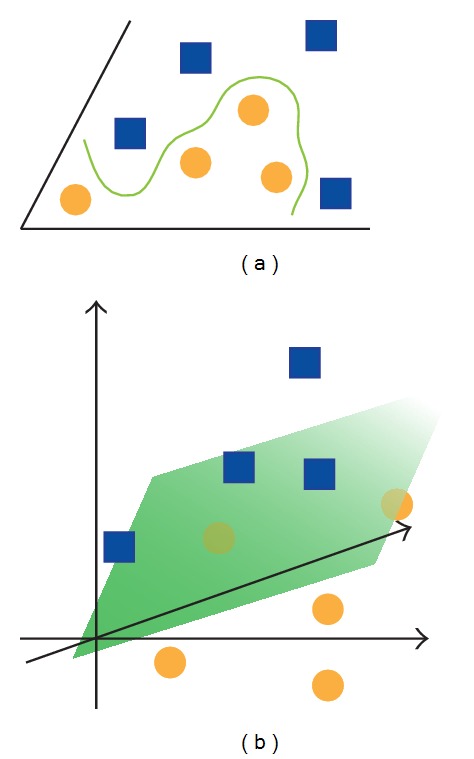
The kernel function maps the data from a certain space into a higher dimensional space where data become linearly separable. In this graphic example, in the bidimensional space, data were not linearly separable into two classes (a). In the three-dimensional space instead (b), they can be separated by a plane.

Classification is performed using the so-called *kernel functions*, which map a nonlinearly separable set of data defined in an *n*-dimensional space, into a higher dimensional space (possibly of infinite dimension) where data become linearly separable; that is, they can be divided by a hyperplane (Figure 1). This linearly separable problem can be solved using SVM.

Many different types of kernel functions have been proposed in the literature (i.e., polynomial, Gaussian radial basis functions, sigmoid functions, etc.). Since the use of a specific kernel function influences the performance of the classification process, it is important to consider several solutions and select the best one. Methods such as boot-strapping [44] and cross-validation are commonly used for kernel selection.

As a rule of thumb, a linear kernel is less prone to overfitting and it is useful for features selection, since it is easy to retrieve a weight associated to each feature. Otherwise, a Gaussian kernel provides generally better performance, but it does not provide a direct estimate of the weights to be assigned to each feature.

The overall classification step can be divided into two phases: *training *and* validation*. In particular, when training an SVM, the user has to decide which kernel to use and a series of parameters describing the SVM and the kernel. Then, given a set of training examples, that is, objects previously marked as belonging to one of the two possible categories, an SVM training algorithm builds a model that will be subsequently used to assign new instances into either one category or the other. Different approaches have been proposed in order to achieve fast training times [45–47]. The classifier is trained by maximizing the margin of separation between the two groups provided with the training set. During the validation, instead, employing the model built during the training phase, the SVM predicts the group to which a new set of previously unseen objects (the testing set or validation set) belongs.

Obviously, training and testing set have to be nonoverlapping. This requires a great amount of data do be acquired. To overcome this problem, a cross-validation technique can be used: the set of all data is split into two subsets, that is, the training and the testing sets. The split is performed several times adopting different partitions, and each time the accuracy value of the obtained classification is recorded. At the end, all the accuracy values are averaged to provide the final accuracy of the classification algorithm.

In the case of a small dataset, a *leave-one-out *cross-validation is commonly adopted [48]: in this case, at each iteration, a single pair of objects (one from each class) is excluded from the overall group, and the classifier is trained using all the other objects. Then the initially excluded pair is used for the validation phase. The overall procedure is iterated for each object pair.

## 3. Methods Developed in Our Laboratory

Given the uprising role SVM is gaining in neuroimaging field, we have been investigating different approaches in order to extract different features from MRI brain data in the last few years [28–34]. In each approach, we started from the evidence that there are brain structural and functional differences between subjects with schizophrenia and healthy controls (HC).

For simplicity, according to the way in which the SVM input is extracted, our studies can be divided into two main groups: one in which each object (i.e., brain) is described by features derived from the object itself and one in which each object is described by distance/dissimilarity measures evaluated by the comparison between pairs of objects. A complete dissimilarity representation provides a square matrix with the dissimilarities between all pairs of objects.

### 3.1. Methods Based on Feature Vectors

This is the classical approach used in pattern recognition and machine learning, and it consists in representing each object to be classified as an *n*-dimensional vector of numerical feature. When representing MRI data, the feature values might correspond to the gray level of each voxel of the acquired volume. In such a way the feature vector encodes either the pattern of brain activation [49], in the case of functional neuroimaging data, or the pattern of gray and white matter volume, in the case of structural data [50].

In our works, several different features have been chosen to represent objects to be classified. One of the advantages of the implemented methods is that registration between subjects is never required, since, as it will be described in the next sections, the features that we chose and that have been extracted are always position and scale invariant.

The study by Castellani et al. [29] focused on one region of interest (ROI) (left amygdala) manually traced on a cohort of 124 subjects (64 diagnosed with schizophrenia plus 60 HC) and characterized by using a local geometric feature, that is, the *shape index*, which encodes the curvatures of a generic surface point, by capturing the intuitive notion of *local shape *[51]. The 3D surface was computed from the set of 2D ROIs as a triangle mesh using marching cubes. Given the definition provided in Koenderink and van Doorn [51], the shape index can take any value in the interval [−1,1], where the values −1 and 1 are high local curvature, and 0 stands for no local curvature (i.e., flat surface). All values extracted for a subject were quantized in a fixed number of bins, and a histogram of occurrences, which represents the descriptor of a given subject, was computed. The subsequent step of the algorithm was based on the research on natural language processing: in particular, the computed quantized shape descriptors were considered as a set of *visual words* from which a generative model was learned. Generative models are built to understand how samples were generated, and they are learned to find local patterns of cooccurrences, by leading to the definition of the *visual topics*. The generative model chosen in Castellani et al. was the probabilistic latent semantic analysis (pLSA) [52]. Two models have been learned, one for each group (controls and patients), to provide a score for each subject. The set of scores was finally used as input for the SVM classifier. In this study, two kinds of kernels have been considered (the histogram intersection kernel and the *χ* ^2^ kernel), and the cross-validation strategy was used to evaluate the classification performances. 75% of the samples were randomly extracted as training set, using the rest for testing, and the overall process was repeated 20 times. The best result in terms of accuracy was 86.13% ± 2.17, obtained with 45 topics and the histogram intersection kernel. It is worth noting that the SVM classification performed directly on the feature histograms (i.e., without the pLSA) by using the same validation strategy and kernel led to an average accuracy of 58.70% ± 9.78. This means that, thanks to the pLSA analysis, a drastic improvement can be obtained in classifying morphological features in schizophrenia.

As further test, in the same study, PCA was used to reduce the dimensionality of the quantized shape index histograms, for different values of the saved components. The classification test was performed using the previously employed kernels. Results for PCA, in terms of accuracy, were always between 50% and 60%, thus demonstrating the superiority of pLSA-based dimensionality reduction.

In Castellani et al. [31], instead of using the shape index, a new shape descriptor based on advanced diffusion geometry techniques was introduced. The work focused on one ROI (left thalamus), manually traced on a cohort of 60 subjects (30 diagnosed with schizophrenia plus 30 HC), from which structural T1-weighted MRI images were acquired. Again, the characteristics of the introduced descriptor allowed avoiding the registration between subjects. In fact, local geometric properties were encoded by the *heat kernel* [53]. This is an isometric invariant which allows describing the geometry of an object by a vector obtained convolving the heat kernel with the object descriptor (in our case the ROI mask volume). The heat kernel was obtained as the solution of the heat equation, which models how heat diffuses as function on time on a shape. Intuitively, *local* shape characteristics are highlighted through the behavior of heat diffusion over *short* time periods, and, conversely, *global* shape properties are observed while considering *longer* periods. So, the variation of one parameter, time, allows characterizing the properties of a shape at different scales. By fixing the number of scales, we built a histogram of local heat kernel values observed at each point of a surface mesh or at each point of a volumetric representation of the ROI. The resulting histogram represented the global heat kernel signature (GHKS), which was then used as input for the SVM classifier. In this study, both surface meshes and volumetric representation were considered.

The *bag-of-words* (*BoW*)* approach* is inspired by well-established methods of indexing and retrieval of text documents. Text documents can be summarized by their word counts, or *bag-of-words*, and by the frequency of occurrence of words drawn from a defined word vocabulary.

In the field of pattern recognition, after having performed feature detection, each object is associated to its signature, that is, its vector of features. In BoW approach, a large sample of features is collected from the set of objects. This large sample is then quantized with some clustering techniques, usually with *k*-means clustering, obtaining a number *k* of clusters. The center of each cluster is called *visual word* or *feature prototype*. The set of all the obtained visual words provides the so-called *feature vocabulary*.

The chosen kernels functions were linear, polynomial (degree = 3), and radial basis function, and the *learning by example* approach was introduced by adopting leave-one-out cross-validation procedure. Finally, the descriptor was compared with the *ShapeDNA* descriptor [54], which, however, does not deal with multiple scales and takes into account only global information. Both the GHKS and the ShapeDNA descriptor produced the best results when the volumetric approach was employed, in combination with the radial basis function kernel, even if the GHKS descriptor was more stable by varying the type of kernel employed. Furthermore, the best result obtained with the ShapeDNA descriptor was 73.33%, while adopting the proposed GHKS descriptor, accuracy was 83.33%.

A different feature extraction method, as well as a new kernel function were proposed in Castellani et al. [28] to study the dorsolateral prefrontal cortex (DLPFC). The ROIs were manually traced on a cohort of 108 subjects (54 diagnosed with schizophrenia plus 54 HC), from which structural T1-w MRI images were acquired. The local description of the ROI was obtained using the scale-invariant feature transform (SIFT) [55]. This is an algorithm that, given its ability in finding distinctive key points that are invariant to location, scale, and rotation, is commonly used in computer vision problems to detect and describe local features in images. Starting from a set of landmarks, which in our case have been extracted employing the difference-of-gaussian (DoG) point detector, the pixels of the landmark's neighbourhood were encoded using SIFT into a multidimensional feature vector which described the local area. In this way, each brain was represented by a set of feature vectors (one for each landmark), but all sets could have different cardinalities, according to the number of extracted landmarks. Then, a second processing procedure was performed in order to allow comparisons among subjects. Primarily, the set of all feature vectors from all brains was clusterized using the *k*-means clustering technique [36]. Subsequently, the centroids of the clusters were considered as *visual words *or* features prototypes* providing a quantization of the feature space, which is called the *feature vocabulary*. This procedure is performed to apply the bag-of-words (BoW) approach [56]. Finally, a weighting function was introduced to define the relevance of the detected visual words, in discriminating between patients and controls. However, although SVM requires as input a set of fixed length vectors, here a subject was represented by a set of local features with variable cardinality. In order to tackle this problem, a suitable kernel function has been employed. Since features are local, such kernel functions are known in the literature as *local kernel* or *matching kernels* [57]. The validation procedure was performed adopting the leave-one-out cross-validation procedure.

The obtained results showed an accuracy improvement when introducing the weighting function. Performances further increased, regarding the whole dataset (left hemisphere 75%, right hemisphere 66.38%), when only females (left side 84.09%, right side 77.27%) or only seniors (left side 81.25%, right side 70.83%) were taken into consideration. This is very interesting since gender and age have significant impact on brain maturation in both healthy subjects and patients with schizophrenia. Therefore, such variables should always be considered in machine learning techniques when analyzing MRI dataset from individuals with schizophrenia to increase the accuracy.

Finally, in Ulaş et al. [33, 34], two variations of the recently proposed multiple kernel learning (MKL) methods [58, 59] were implemented. As we have already remarked, selecting the kernel function and its parameters is an important issue in training. MKL methods, instead of learning a specific kernel for all features, use a combination of different kernel functions, one for each feature extracted from the data. With this approach, each kernel function contributes proportionally to an assigned weight parameter to the final space transformation, and it becomes possible to integrate and select the contribution from different parts and different features of the brain. The difference among most MKL algorithms is the optimization method which is applied to estimate the weights or the combination rule used [58, 60].

The simplest way is to combine the kernels as a weighted sum which corresponds to the linear MKL. In the literature, different methods have been proposed. For instance, the rule-based MKL (RBMKL) algorithm, that trains an SVM by means of the combined kernels [61], the group Lasso based MKL (GLMKL) algorithms [62], or the iterative simple MKL (SMKL) algorithm [63], which uses projected gradient updates and trains single-kernel SVMs at each iteration. In particular, in Ulaş et al. [33] the weights computed by MKL method were used in order to highlight the importance of each brain part and feature in the detection of the disease. In Ulaş et al. [34], instead, the MKL approach was exploited to introduce a priori information linked to patients' covariates in order to improve the classification accuracy.

In the former study [33], four pairs of ROIs (left and right amygdala, left and right entorhinal cortex, left and right superior temporal gyrus, and left and right thalamus) were manually outlined from a T1-weighted MRI scan acquired from a dataset of 100 subjects (50 diagnosed with schizophrenia plus 50 HC). In this work, three different descriptors have been computed. The first descriptor was represented by gray level tissue distribution (i.e., histograms) evaluated in each ROI after MRI scale standardization based on landmark matching [64]. The remaining descriptors represented two geometric features, that is, the shape index and the curvedness, evaluated at each vertex of a triangle mesh computed from the set of 2D ROIs using marching cubes. Values were then quantized in order to build a histogram of occurrences for both kinds of geometric properties. The three obtained descriptors were used as input for the SVM. To assess the performance of the proposed methodology, we used a leave-one-out cross-validation strategy.

The accuracies of combining ROIs for each descriptor have been evaluated for a group of classifiers. Among these, a new brain classification method, the clustered localized multiple kernel learning (CLMKL), has been introduced. In localized MKL (LMKL) [65], a decision function is defined; its parameters depend on the input data, that is, localized information. The CLMKL approach, instead of letting the algorithm choose the partitioning, exploited a priori partitioning based on expert knowledge; for instance, subjects can be clusterized into males and females. CLMKL thus learns a separate combination of input kernels for each cluster. Such combination of individual base classifiers is driven by the *gating function* method [66], which has to be properly formulated in order to incorporate the desired a priori information, that is, the proposed GLMKL, the single kernel SVM, the SVM on the concatenation of ROIs, the rule-based MKL, and the simple MKL. In general, our study revealed that MKL methods were better than single kernel SVMs, and the best result in terms of accuracy (81%) was obtained using either GLMKL or simple MKL. Finally, combining all the feature sets and all the ROIs, there was a further improvement in the accuracy (84%), thus suggesting the importance of combining different ROIs and multiple descriptors.

In Ulaş et al. [34], three pairs of ROIs (left and right DLPFC, left and right entorhinal cortex, and left and right thalamus) were manually outlined from a T1-weighted MRI scan acquired from a dataset of 82 subjects (42 diagnosed with schizophrenia plus 40 HC). The rationale of the work originated from the evidence that in brain classification there is a general diversity of the brain properties in accordance with gender and age, as previously shown by our group [28]. The CLMKL was used in order to explicitly encode the known intraclass variability into the classification model. Since in CLMKL approach a priori partitioning based on expert knowledge is exploited, subjects were clusterized into males and females. In our study [33] the gating function had to behave differently regarding gender. The gating model parameters were computed using alternating optimization. First, the kernel weights were fixed and the SVM parameters were estimated by standard solvers. Second, the SVM parameters were fixed and kernel weights were estimated by a gradient descent procedure.

Classification was performed both at a single-ROI value and combining all considered ROIs.

In the former case, different classifiers have been compared, that is, the proposed CLMKL, the standard SVM applied on the feature set, the concatenation of the feature and the gender information, and the localized MKL. Linear kernels have been used in all experiments, together with a leave-one-out validation scheme [33]. CLMKL resulted to be the most accurate method every time, with a maximum accuracy of 81.71% when considering the left entorhinal cortex. In the latter case, that is, when combining all the traced ROIs, CLMKL was compared to the already cited methods, plus the rule-based MKL and the simple MKL. Once again, CLMKL proved to be the most accurate method, with an accuracy of 90.24%. Other methods increased their accuracy if data were divided into male/female subsets (e.g., SVM increased from 71.95% to 75.00% and 76.19% when classifying male and female, resp.), but the improvements did not reach CLMKL's results.

### 3.2. Methods Based on Pairwise Dissimilarities

In methods based on vector of features, each instance to be classified is described in an absolute way, that is, disregarding any comparison with other objects. In the dissimilarity approach, instead, instances are described using pairwise dissimilarities to a representation set of objects [67]. Such a method might appear closer to what is commonly done by human beings in everyday life. Intuitively, when we are required to classify objects into groups, we typically proceed by comparison; that is, we describe each object in relation to all the others, rather than in an absolute way. It can be easily derived that the choice of the dissimilarity measure is crucial in this approach and must properly characterize the data analyzed in the study. For example, when histograms are used to describe the data, a proper dissimilarity measure can be the histogram intersection or the earth mover's distance.

In the context of automatic classification, the dissimilarity representation is transformed into a vector space in which traditional statistical classifiers can be used. Unlike the related kernel approach, whose application is often restrained by technicalities like fulfilling Mercer's condition, basically any dissimilarity measure can be used [68]. For instance, in the field of biomedical imaging, a common feature used to describe images is the registration error, that is, the transformation that best aligns an image to another one. Though this measure can have an intuitive meaning (the registration error will be small when two images are similar and vice versa), it does not fulfill Mercer's condition [9], so it could not be used within a classic approach.

We applied the dissimilarity approach in two different studies [30, 32], where 7 pairs of ROIs (for right and left hemispheres resp.) were considered, that is, amygdala, DLPFC, entorhinal cortex, Heschl's gyrus, hippocampus, superior temporal gyrus, and thalamus.

In Ulaş et al. [30], gray value histograms and their probability density functions (pdfs) were obtained for each ROI in 124 subjects (64 with diagnosed schizophrenia plus 60 HC), from which a T1-weighted MRI sequence had been acquired, while in Ulaş et al. [32] two different MRI modalities were used, that is, T1-weighted and DWI sequences, both acquired from a cohort of 114 subjects (59 with diagnosed schizophrenia plus 55 HC). Since ROIs were traced on T1-weighted scans, in the latter study a coregistration step was needed in order to properly realign each ROI from the T1-weighted space into the DWI space. Besides, for each of the 14 ROIs, together with T1-weighted gray value histograms and their pdfs, three additional parameters were extracted, that is, two geometric shape descriptors from the T1-weighted volume, that is, the *shape index* and the *average curvature*, both evaluated at each vertex of a 3D surface computed from the set of 2D ROIs as triangle mesh using marching cubes (histograms and pdfs were obtained) and the histogram and pdf of water apparent diffusion constant (ADC) from the DWI volume.

In both studies, 13 dissimilarity measures were used, for example, for pairs of histograms: Euclidean distance, *L*1 distance, intersection, diffusion distance, *χ* ^2^ distance, and Earth mover's distance; for pairs of pdfs: Euclidean distance, *L*1 distance, Earth mover's distance, Bhattacharyya distance, symmetrized Kullback-Leibler divergence, original asymmetric Kullback-Leibler divergence, and Jensen-Shannon divergence. The choice of the dissimilarity measures strongly depends on the considered data, and it is not in the aim of our paper to provide guidelines to decide the most proper choice. However, we suggested some examples in the paper to help the reader to understand the basic concepts of the dissimilarity measure approach.

Involved dissimilarity measures were not necessarily Euclidean measures. When comparing a pair of histograms, a distance between them could be computed using the norm function. Anyway, since the norm was not the most proper way to characterize histogram differences, we defined a distance between histograms based on the histogram intersection.

In the dissimilarity space basically any traditional classifier could be used. The number of dimensions equaled the number of objects, that is, the number of subjects of the dataset. If such dataset was large, many classifiers would need dimension reduction techniques or regularization to work properly. SVM allowed avoiding the dimension reduction.

In both papers the number of dissimilarities matrices was given by the product of the number of ROIs (14), the number of dissimilarity measures (13), and the number of modalities considered (1 in [30] and 4 in [32]).

Classification was performed both at a single-ROI level (i.e., considering only one ROI) and at a multi-ROI level (i.e., combining all considered ROIs).

For each test we evaluated the leave-one-out error. Two classifiers were considered, the 1-nearest neighbour (NN) rule on the original dissimilarities and the linear SVM in dissimilarity space. The last one avoided complications that could arise from the measures being non-Euclidean. At a single-ROI level, the leave-one-out error estimated for the linear SVM in dissimilarity space proved to be lower than the error estimates for NN using the original dissimilarities (standard approach).

Once again, when combining all ROIs at the same time, the classification on the dissimilarity space outperformed the standard approach. Moreover, the multi-ROI approach brought a drastic improvement by confirming the complementary information enclosed onto the different brain subparts. Finally, the error estimates were computed on the overall dissimilarity matrix (for all the measures and ROIs) for both standard approach and dissimilarity-based approach, respectively, yielding the best results (79%).

In Ulaş et al. [32], in addition to the 1-nearest neighbour (NN) rule on the original dissimilarities and the linear SVM in dissimilarity space, linear SVM classifier on the original feature space was employed. Besides, in addition to classification performed at a single-ROI level and combining all considered ROIs, a further classification was obtained combining different MRI modalities.

In the single-ROI classification, regardless of the considered feature, SVM classifier in the dissimilarity space was always better than classifiers in the standard space. The best accuracy (78.07%) was obtained on the left amygdala, using the histogram of intensities and the dissimilarity measure of Bhattacharyya distance.

In the multi-ROI classification, obtained combining all ROIs but fixing both the modality and the dissimilarity measure, the proposed classification in the dissimilarity space provided better results than the standard approach. The maximum of accuracy was obtained using the *L*1 distance between pdfs as dissimilarity measure, and it reached 76.32%. Moreover, on average, the multi-ROI approach provided an improvement regarding single-ROI approach, thus confirming the complementary information enclosed onto the different brain subparts. Finally, in the multimodal classification, the information gathered from the two different MRI sequences (i.e., T1-weighted and DWI) was combined. In particular, to get the best result, the most accurate four ROI-dissimilarity pairs from each modality were chosen; then an exhaustive search on the combination of these matrices was performed. The best accuracy (86.84%) was obtained with the combination of two dissimilarity matrices from intensities (both from left DLPFC, one using original asymmetric KL divergence and one using Bhattacharyya distance between pdfs) and one dissimilarity matrix from shape index (from right DLFPC, using *χ* ^2^ distance between histograms). Applying the same methodology, the best results reached with 1-NN and with SVM on the original feature space were limited to, respectively, 76.32% and 83.33%.

Overall, these two studies highlighted the complementary information enclosed in the combination of several ROIs: the fusion of information from various regions of the brain allows improving the results obtained with the single-ROI approach. However, this is not the case for multimodal MRI information. Indeed, adding DWI data to structural T1-weighted sequences did not reveal to be useful in our research for obtaining more accurate results.

## 4. Other Recently Proposed Methods

During the last few years, the number of studies which applied SVM in order to investigate psychiatric disorders has been increasing. To get a glimpse of different proposed techniques, we cite some original recent studies [23, 25, 35] which have been applied to schizophrenia, and we compare their results in terms of accuracy.

The study by Fan et al. [23] used a pattern classification method for the identification of structural brain abnormalities based on regional tissue volumetric information. In order to perform a quantitative comparison of different individual brain images, warping each image into a template space was necessary. The study was performed on two groups, a female dataset (dataset A, 23 subjects diagnosed with schizophrenia and 38 healthy controls) and a male dataset (dataset B, 46 subjects diagnosed with schizophrenia and 41 healthy controls). The diagnostic accuracy reached in distinguishing individuals with schizophrenia from healthy controls was 90.2% for the female dataset and 90.8% for the male dataset.

The study by Koutsouleris et al. [25] represented a first attempt in identifying individuals in different at-risk mental states (ARMS) of psychosis. The aim of the study was to verify whether it was possible to detect early a psychosis during its prodromal phase. The study was performed on a cohort of 45 individuals with ARMS, plus a corresponding group of healthy controls. The novelty of this study was related to the use of multivariate neuroanatomical pattern classification in order to evaluate the feasibility of early recognition and disease prediction in individuals with ARMS. The diagnostic accuracy reached in distinguishing individuals with ARMS from healthy controls was between 78% and 94%.

A hybrid machine learning method has been proposed in the study by Yang et al. [35], in order to classify schizophrenia patients and healthy controls. In this study, two SVMs were applied, one on MRI data and one on single nucleotide polymorphism data, and then they were combined together. The method was applied on 20 patients and 20 healthy controls, and it provided a classification accuracy of 87%.

Unlike the methods developed in our laboratory, all of them performed a warping of the data, that is, each brain volume was registered to a brain template (e.g., the Montreal Neurological Institute (MNI) template), in order to compensate for the interindividual anatomical variation. Moreover, a limitation of these studies was the relatively limited sample size: all the authors asserted that results should be replicated on larger populations. The results, in terms of accuracy, of these three studies are summarized in Table 2.

## 5. Conclusions

In recent years the interest of the scientific community in computational neuroscience is constantly growing [69]. In particular, computational methods have been increasingly applied to the field of magnetic resonance imaging (MRI) after processing [70]. The aim is to analyze MRI data by the means of innovative bioinformatic methods in order to detect and describe human brain features.

Neuroimaging studies exploiting MRI have revealed structural and functional alteration in schizophrenia. Nevertheless, these findings have not been extensively applied to clinical practice, so far, to help in the diagnosis and treatment of this psychiatric disorder.

In this study, we have described recent support vector machine-based methods developed and applied in psychiatric neuroimaging by our research group (results are summarized in Table 1). Each of the developed methods focused on specific regions of interest in the brain, in order to study whether it was possible to define a set of features that could be discriminative in the diagnosis of schizophrenia.

The results obtained by studies using machine learning are encouraging. They have shown that exploiting complementary information, coming either from various regions of the brain or from the use of different features, allows reaching extremely promising levels of accuracy in classifying healthy controls and patients affected by schizophrenia. These results suggest that the application of machine learning techniques to neuroimaging studies will potentially be of help in automatically classifying patients with schizophrenia on the basis of MRI, with a potential tremendous translational impact.

As shown in our works, classification in medical environment is extremely variable: different data may be available, different brain districts may be considered, and some measures may be useful to describe some medical conditions but they may be useless in different others. This makes it extremely difficult to suggest the best analysis pipeline and classifier to be used. The planning step plays an important role in studies based on classification methods, and different approaches should always be considered and tested.

However, although nowadays we are far away from using automatic image-based classification techniques to make a diagnosis, in the long run, they might help clinicians in reliable and early detection of affected patients, potentially becoming a crucial tool for the real world of psychiatric practice. Indeed, these methods could be added to the conventional diagnostic process as a complementary assessment for the evaluation of brain anatomy and morphometry in patients suffering from schizophrenia. This would represent a major clinical advantage, together with a translational scientific approach, to the diagnosis of schizophrenia. Further studies are now necessary to improve the potential contribution of computational methods applied to MRI in the early stages of schizophrenia, for instance, by including subjects at risk to develop the disease or examining in depth the effects of specific covariates, such as age, gender, and ethnicity.

Finally, in addition to providing a potential help in diagnosis, machine learning may offer some information for the treatment and prognosis of the illness. For instance, applying these techniques by considering MR data with other variables such as pharmacological treatment will allow classifying subjects responding to specific drugs.

## Figures and Tables

**Table 1:** Performance evaluation.

Author	Sample size	MRI technique	SVM input	Number of ROIs	Best accuracy (%)
[29]	SCZ = 64 HC = 60	Structural T1-w	Features vector	1 (left Amy)	86.13
[30]	SCZ = 64 HC = 60	Structural T1-w	Pairwise dissimilarities	7l + 7r (Amy, DLPFC, EC, HG, Hipp, STG, and Tha)	79
[31]	SCZ = 30 HC = 30	Structural T1-w	Features vector	1 (left Tha)	83.33
[32]	SCZ = 59 HC = 55	Structural T1-w DWI	Pairwise dissimilarities	7l + 7r (Amy, DLPFC, EC, HG, Hipp, STG, and Tha)	86.84
[33]	SCZ = 42 HC = 40	Structural T1-w	Features vector	3l + 3r (DLPFC, EC, and Tha)	90.24
[28]	SCZ = 54 HC = 54	Structural T1-w	Features vector	1 (DLPFC)	84.09
[34]	SCZ = 50 HC = 50	Structural T1-w	Features vector	4l + 4r (Amy, EC, STG, and Tha)	84

Each method compared subjects affected by schizophrenia (SCZ) with healthy controls (HC). All methods focused on specific regions of interest (amygdala (Amy), dorsolateral prefrontal cortex (DLPFC), entorhinal cortex (EC), Heschl's gyrus (HG), hippocampus (Hipp), superior temporal gyrus (STG), and thalamus (Tha)). The last column shows the performance of each algorithm in terms of accuracy, which is the overall proportion of correct classification (i.e., the number of correctly classified subjects divided by the number of all subjects).

**Table 2:** Comparison between three state-of-the-art studies. Performance evaluation.

Author	Sample size	MRI technique	Best accuracy (%)
[23]	SCZ = 23 (A) 46 (B) HC = 38 (A) 41 (B)	Structural T1-w	90.2 (A) 90.8 (B)
[25]	ARMS = 45 HC = 75	Structural T1-w	78 ÷ 94
[35]	SCZ = 20 HC = 20	fMRI	87

In the studies in the first and last rows, SVM was used to compare subjects affected by schizophrenia (SCZ) with healthy controls (HC). The study in the second row applied SVM to identify individuals in different at-risk mental states (ARMS) of psychosis. The last column shows the performance of each algorithm in terms of accuracy.
